# The War on the Hudson

**Published:** 1884-03

**Authors:** 


### THE WAR ON THE HUDSON.

The New York State Medical Society at their last meeting, held February 5th, again ratified the *New Code.* This ratification of liberal principles so angered the old fogies—who could not be gentlemanly or honest in their conduct unless bound by an oath—that they (the old fogies) rebelled, seceded, and formed an iron-clad, double-riveted, oath-bound society! Seventy-five joined this Protective Union.

